# Oral manifestations and dental management in patients undergoing CAR-T cell therapy: a retrospective observational study

**DOI:** 10.1007/s00520-026-10824-6

**Published:** 2026-06-06

**Authors:** Fernanda de Paula Eduardo, Luiza Deitos Menti, Mariana Henriques Ferreira, Danielle Lima Correa de Carvalho, Cristina de Paula Novaes, Beatriz Helena da Silva Martins, Lucila Nassif Kerbauy, Nelson Hamerschlak, Letícia Mello Bezinelli

**Affiliations:** 1 Faculdade Israelita de Ciências da Saúde Albert Einstein, São Paulo, Brazil; 2 Hospital Israelita Albert, São Paulo, Brazil

**Keywords:** CAR-T cell therapy, Oral complications, Supportive oral care, Hematologic malignancies, Immunotherapy

## Abstract

Chimeric antigen receptor T-cell (CAR-T) therapy has emerged as a highly effective treatment for refractory and relapsed hematologic malignancies. Despite its clinical benefits, CAR-T therapy is associated with a unique toxicity profile, including cytokine release syndrome (CRS), immune effector cell–associated neurotoxicity syndrome (ICANS), and prolonged immunosuppression. Oral complications in this population remain underreported. This retrospective case series was aimed at describing oral manifestations and dental management in patients undergoing CAR-T therapy at a tertiary referral center. Medical and dental records of ten patients treated with CAR-T therapy between 2024 and 2025 were reviewed. Demographic data, underlying hematologic diagnoses, CAR-T-related toxicities, and oral findings were analyzed descriptively. The main oral manifestations observed were xerostomia, opportunistic infections, dental pain, and dysgeusia. Oral care focused on symptom control, prevention of secondary infections, and supportive oral management during periods of severe immunosuppression. These findings suggest that early dental involvement may contribute to supportive care strategies, particularly for the detection and management of opportunistic infections and dental infectious foci in neutropenic patients. Standardized oral assessment protocols and preventive dental measures may contribute to supportive clinical management and symptom monitoring in this patient population.

**Supplementary Information:**

The online version contains supplementary material available at 10.1007/s00520-026-10824-6.

## Introduction

Chimeric antigen receptor T-cell (CAR-T) therapy is a recently developed and innovative treatment for relapsed and refractory hematologic malignancies, including B-cell acute lymphoblastic leukemia, B-cell non-Hodgkin lymphoma (NHL), chronic lymphocytic leukemia (CLL), and multiple myeloma [2, 7]. CAR-T therapy is a form of cellular immunotherapy that involves ex vivo genetic modification of T lymphocytes to express a chimeric antigen receptor (CAR). These CAR-T cells are capable of targeting overexpressed surface antigens on tumor cells and become activated independently of major histocompatibility complex (MHC) recognition, ultimately resulting in tumor cell destruction [3].

More recently, studies have explored the use of donor-derived (allogeneic) CAR-T cells; however, CAR-T products are typically manufactured from the patient’s own T cells (autologous) [5]. Prior to infusion of the genetically modified T cells, patients undergo a lymphodepleting chemotherapy regimen, most commonly consisting of cyclophosphamide and fludarabine. This lymphodepletion not only facilitates the expansion and persistence of infused CAR-T cells but also reduces the likelihood of host immune rejection [4, 6].

Currently approved CAR-T therapies have demonstrated excellent response rates across different hematologic malignancies. Overall response rates ranging from 44 to 91% and complete response rates between 28 and 68% have been reported in patients with B-cell lymphoma and chronic lymphocytic leukemia. Among patients with B-cell acute lymphoblastic leukemia treated with CAR-T therapy, complete response rates range from 62 to 86%. In multiple myeloma, response rates of 73 to 100% have been observed, with complete response rates varying between 33 and 83% [7].

Despite these encouraging outcomes, CAR-T therapy is associated with significant treatment-related toxicities, particularly cytokine release syndrome (CRS) and immune effector cell–associated neurotoxicity syndrome (ICANS) [20]. The most common complication is cytokine release syndrome (CRS), a systemic inflammatory response caused by excessive activation of CAR-T cells along with other immune cells, including macrophages and dendritic cells [9, 21]. Common clinical manifestations of CRS include fever, myalgia, and hypotension. Although an appropriate degree of CRS may be associated with enhanced CAR-T efficacy, inadequate management can lead to hemodynamic instability and multiorgan failure [8].

Another serious adverse effect is immune effector cell–associated neurotoxicity syndrome (ICANS). Approximately 67% of patients with leukemia and 62% of patients with lymphoma develop some degree of ICANS (Sterner & Sterner, 2022). Clinically, ICANS may present with headache, confusion, attention deficits, seizures, and transient coma.

Oral mucositis is a common complication in patients undergoing hematopoietic stem cell transplantation (HSCT), particularly among those receiving myeloablative conditioning regimens, due to the high mucosal toxicity associated with intensive chemotherapy and radiotherapy. In contrast, patients treated with CAR-T therapy generally do not develop oral mucositis with the same frequency or severity, as this treatment does not require intensive conditioning regimens that directly damage the oral mucosa. Furthermore, the mechanism of action of CAR-T cells is more closely related to immunomodulation of the tumor microenvironment and targeted cytotoxicity, rather than the nonspecific mucotoxic effects typically observed with alkylating agents or total body irradiation used in HSCT [10, 12, 13, 25]. Nevertheless, weight loss has been reported during CAR-T therapy and may negatively impact prognosis and overall survival. Oral alterations such as dysgeusia and xerostomia may further exacerbate this condition by impairing oral intake [14].

Although CAR-T cell therapy is rapidly expanding in clinical oncology, its potential oral adverse effects and the appropriate management of oral care during treatment remain poorly described in the literature. This study is aimed at reporting a series of ten cases of patients undergoing CAR-T cell therapy, with a specific focus on oral manifestations and supportive oral care measures implemented during treatment.

## Materials and methods

Ten hospitalized patients who underwent CAR-T cell therapy between January 2024 and July 2025 at a single tertiary hospital in Brazil were followed by the dental team from the pre-hospitalization phase, through lymphodepletion, until hospital discharge and were included in this retrospective case series. Three patients with multiple myeloma were enrolled in the CARTITUDE protocol; one patient with chronic lymphocytic leukemia (CLL), three with B-cell acute lymphoblastic leukemia (B-ALL), and one with diffuse large B-cell lymphoma (DLBCL) were included in the CARTHIAE protocol; and two patients with DLBCL were treated under the Yescarta protocol.

All patients underwent a comprehensive dental evaluation prior to initiation of CAR-T cell therapy to identify potential sources of acute infection or other oral cavity abnormalities. Patients received standardized oral care instructions throughout treatment, including oral hygiene with a soft-bristled toothbrush and the use of an enzymatic mouthwash, both performed three times daily. Artificial saliva was prescribed in cases where xerostomia was reported. These oral care measures were reinforced by the dental team during follow-up consultations.

Clinical and laboratory data were retrospectively extracted from the hospital’s electronic medical records and included variables such as age, hematologic diagnosis, lymphodepleting regimen, medical comorbidities, daily complete blood count, dietary tolerance, presence or absence of diarrhea, and the occurrence and severity of cytokine release syndrome (CRS) and immune effector cell–associated neurotoxicity syndrome (ICANS).

Additional variables were collected during scheduled dental consultations conducted twice a week, during which patients responded to binary (yes/no) questions assessing the presence of xerostomia and taste alterations. Clinical oral examinations further complemented these assessments to investigate any oral changes, including mucositis and opportunistic infections.

### Ethical considerations

This retrospective case series was approved by the Institutional Research Ethics Committee of the hospital in which the study was conducted (CAAE: 94925125.9.0000.0071). The study was conducted in accordance with the ethical principles outlined in the Declaration of Helsinki. Given the retrospective and observational nature of the study, with no additional interventions beyond standard clinical care, the requirement for informed consent was waived by the Ethics Committee.

## Results

### Patient characteristics

This retrospective case series included ten patients treated with CAR-T cell therapy between 2024 and 2025, comprising individuals diagnosed with diffuse large B-cell lymphoma (DLBCL) (*n* = 3), multiple myeloma (MM) (*n* = 3), B-cell acute lymphoblastic leukemia (B-ALL) (*n* = 3), and chronic lymphocytic leukemia (CLL) (*n* = 1). Patient ages ranged from 23 to 80 years, with a predominance of males (*n* = 8) compared with females (*n* = 2).

All patients received a lymphodepleting regimen consisting of cyclophosphamide and fludarabine, followed by a two-day rest period prior to CAR-T cell infusion. Dosages were adjusted according to individual comorbidities. Reported comorbid conditions included hypothyroidism (*n* = 3), dyslipidemia (*n* = 2), hypertension (*n* = 2), diabetes mellitus (*n* = 2), deep vein thrombosis (*n* = 1), atrial fibrillation (*n* = 1), hyperuricemia (*n* = 1), hepatitis B (*n* = 1), and gout (*n* = 1). Four patients had no reported comorbidities.

At the time of analysis, nine patients remained under follow-up: eight patients showed no evidence of disease relapse; one patient experienced relapse. One patient died due to sepsis secondary to a pulmonary infection seven months after CAR-T therapy.

#### Adverse events

Although CAR-T cell therapy has demonstrated promising remission rates—both in this study and in the existing literature—it is frequently associated with systemic adverse events. Gastrointestinal symptoms, such as diarrhea, were reported in 20% of patients of this cohort.

Cytokine release syndrome (CRS) occurred in 90% of cases, with grade 1 CRS observed in 70% of patients, grade 2 in 10%, and grade 3 in 10%. The most common CRS manifestations included fever and hypotension, while only one patient remained asymptomatic.

Immune effector cell–associated neurotoxicity syndrome (ICANS) was diagnosed in 40% of the patients. Two patients presented with grade 2 neurological manifestations, including mental confusion and aphasia, while two developed grade 4 neurotoxicity characterized by seizures and coma, requiring admission to the intensive care unit.

Daily hematologic parameters were closely monitored and incorporated into clinical decision-making. Episodes of leukopenia, neutropenia, and thrombocytopenia were frequent, reinforcing the need for continuous oral surveillance throughout the treatment course.

#### Oral adverse events

A total of 69 dental consultations were performed for the 10 patients, each including an oral examination and screening for treatment-related oral symptoms. No cases of oral mucositis were observed, and only one patient reported oral pain during treatment. This patient experienced diffuse dental discomfort attributed to bruxism, which was effectively managed with analgesics and did not recur the following day.

Regarding opportunistic infections, a single case of oral pseudomembranous candidiasis was documented on D + 8 during the period of neutropenia and was successfully treated with topical nystatin.

Xerostomia was reported by five patients (50%) as a transient symptom during CAR-T cell therapy, occurring between days + 6 and + 27 with total resolution after the hospital discharge. Four patients (40%) did not report xerostomia at any point during follow-up, while one patient (10%) presented with preexisting xerostomia attributed to prior therapy, with no changes after CAR-T therapy. To prevent and manage this adverse effect, a xylitol-based mouthwash, which may stimulate salivary flow and alleviate symptoms of dry mouth, was prescribed throughout the hospitalization period.

With respect to dysgeusia, 80% of patients reported no taste alterations. One patient (10%) developed dysgeusia between days + 6 and + 13 after initiation of CAR-T cell therapy, while another patient (10%) presented with dysgeusia prior to treatment, likely related to previous therapy. These taste alterations were managed through discussions between the dental team and the multidisciplinary team, particularly the nutrition team.

Dietary intake data were retrospectively extracted from medical records, revealing variability among patients. Three individuals followed a general diet with reduced appetite, three maintained a standard general diet, and three reported nausea despite being on a general diet. One patient required enteral nutrition via a nasoenteral tube due to insufficient oral intake. These findings underscore the importance of individualized nutritional monitoring, particularly in the presence of gastrointestinal symptoms or compromised oral intake.

## Discussion

CAR-T cell therapy has emerged as a promising approach for the treatment of refractory hematologic malignancies [7]. However, the preceding lymphodepletion regimen, most commonly consisting of fludarabine and cyclophosphamide, may induce significant oral adverse events, such as taste alterations (dysgeusia) and xerostomia, which negatively impact quality of life and treatment adherence.

Cyclophosphamide, an alkylating agent, has been shown to cause direct cytotoxic effects on taste papillae, leading to inflammation and loss of gustatory sensory cells. Studies in murine models have demonstrated that cyclophosphamide administration results in inflammation of taste buds and reduced cellular renewal, contributing to dysgeusia observed in patients undergoing chemotherapy [15].

In addition, chemotherapy can induce both quantitative and qualitative changes in saliva, resulting in increased viscosity and decreased salivary flow. Previous studies have demonstrated that chemotherapeutic agents may affect the salivary glands by inducing ductal dilation in minor glands and promoting degeneration of acinar cells [11]. Reduced salivary flow not only causes discomfort but also compromises oral mucosal lubrication and protection, increasing the risk of infections, dental caries, and difficulties with food intake. The association between xerostomia and dysgeusia is particularly concerning, as both conditions may lead to reduced dietary intake, malnutrition, and overall deterioration of the patient’s general health status (Galanina & Nolden, 2022). Clinical studies indicate that patients who develop xerostomia during chemotherapy are more likely to report taste alterations, suggesting a direct relationship between these manifestations [18].

The involvement of the dental team plays a crucial role in the multidisciplinary care of oncology patients, contributing significantly to patient safety and overall well-being [22]. It is particularly essential in the management of oral complications related to CAR-T cell therapy, continuous monitoring during therapy, and post-treatment follow-up. Adequate oral preparation prior to CAR-T therapy initiation is a fundamental step in reducing the risk of infectious and inflammatory complications during periods of profound immunosuppression and cytopenias [23]. The presence of active oral infectious foci, such as deep carious lesions, periodontitis, or periapical pathology, may serve as entry points for opportunistic microorganisms in immunocompromised patients, increasing the likelihood of severe systemic adverse events [19]. Therefore, comprehensive dental evaluation should be performed before initiation of the lymphodepletion regimen, with emphasis on identifying and eliminating potential sources of infection, providing oral hygiene instruction, and performing dental prophylaxis. In addition, restorative procedures, extractions, and prosthetic adjustments should be completed in advance, allowing sufficient healing time [16]. This preventive approach contributes to maintaining oral health stability during CAR-T cell therapy and reduces the risk of infections.

Furthermore, continuous monitoring by an oral healthcare team, with scheduled visits throughout CAR-T therapy, is essential for early detection of oral alterations and timely intervention [16, 17]. A high frequency of evaluations reduces the risk of progression from initially mild conditions to severe complications and facilitates differentiation between CAR-T–related toxicity and opportunistic infections. Adverse effects such as xerostomia and opportunistic lesions can be assessed and managed during treatment through the use of enzymatic mouthwashes, artificial saliva, topical antifungal therapy, and photobiomodulation when indicated. Early intervention may improve treatment adherence, nutritional intake, and overall quality of life.

Despite strong evidence that oral and dental assessments combined with appropriate interventions can improve quality of life in oncology patients, fewer than 50% receive this type of care prior to initiating chemotherapy [24].

In the present cohort, regular dental monitoring allowed early identification and management of opportunistic infections and oral symptoms, potentially preventing progression to more severe oral complications during neutropenia.

Importantly, given the limited number of reports in the literature addressing oral adverse effects of CAR-T therapy, the present study provides valuable insights into this emerging treatment modality for cancer patients. Systematic documentation of oral manifestations throughout treatment also contributes to the development of a robust clinical knowledge base, supporting real-time therapeutic decision-making and promoting closer integration with the medical care team.

This study has limitations, including its retrospective design, the small sample size, the lack of validated patient-reported outcome measures for xerostomia and dysgeusia, and the heterogeneity of the underlying hematologic diseases and CAR-T protocols. Consequently, the ability to quantify symptom severity longitudinally was limited.

## Conclusion

This retrospective case series provides novel and clinically relevant insights into oral adverse events associated with CAR-T cell therapy. Although no cases of oral mucositis were observed, symptoms such as xerostomia and dysgeusia were frequently reported and may significantly compromise oral intake and nutritional status. In addition, comprehensive dental evaluation prior to treatment initiation is essential to prevent the exacerbation of latent oral infections during the neutropenic period associated with CAR-T cell therapy.

These clinical considerations highlight the importance of incorporating dental professionals as integral members of the multidisciplinary team involved in CAR-T cell therapy. Given that this study is among the first to specifically address the oral implications of this emerging therapeutic modality, further research is warranted to strengthen and expand the current evidence base and to support the development of standardized oral care protocols for patients undergoing CAR-T therapy.

## Supplementary Information

Below is the link to the electronic supplementary material.

ESM 1 (DOCX 10.8 KB)

## Data Availability

"Data is available for consultation upon request".
