# Supplementary material for: Oral manifestations and dental management in patients undergoing CAR-T cell therapy: a retrospective observational study
Source: Support Care Cancer. 2026 Jun 6;34(7):621. doi: 10.1007/s00520-026-10824-6 (PMC13242456; doi:10.1007/s00520-026-10824-6)
Supplement: Supplementary file 1 — (DOCX 10.8 KB) [file 520_2026_10824_MOESM1_ESM.docx]

Supplementary Table S1: Patient Characteristics

| Patient | Sex | Age | Diagnosis | Lymphodepletion | Comorbidities | Dysgeusia | Xerostomia | Oral Mucositis | Oral pain/  secondary infection | Diarrhea | ICANS | CRS | Diet | Follow-up |
| --- | --- | --- | --- | --- | --- | --- | --- | --- | --- | --- | --- | --- | --- | --- |
| 1 | F | 66 | LLC | Fludarabine 25 mg/m² (25% dose reduction due to chronic kidney disease) from D−5 to D−3 + cyclophosphamide 60 mg/kg on D−6. | Hypothyroidism, dyslipidemia, atrial fibrillation secondary to BTK inhibitor therapy, abdominal thrombosis, and macular degeneration, CKD | No | Yes | No | No | No | No | Yes (grade 1) | General/decreased appetite | Death due to sepsis secondary to pulmonary infection |
| 2 | F | 78 | MM | Fludarabine 24 mg/m² from D−5 to D−3 + cyclophosphamide 300 mg/m² from D−5 to D−3, followed by a 2-day rest period. | Diabetes mellitus, hypertension, venous thromboembolism | No | No | No | No | No | No | Yes (grade 1) | General | No evidence of relapse |
| 3 | M | 73 | MM | Fludarabine 30 mg/m² + cyclophosphamide 300 mg/m² on D−5, D−4, and D−3, followed by a 2-day rest period | Hypertension, hypothyroidism, dyslipidemia. | No | Yes | No | No | No | No | Yes (grade 1) | General/decreased appetite | No evidence of relapse |
| 4 | M | 57 | DLBCL | Fludarabine 30 mg/m² + cyclophosphamide 300 mg/m² on D−5, D−4, and D−3, followed by a 2-day rest period. | Diabetes mellitus,  Arthritis | No | No | No | No | No | No | Yes (grade 1) | General/decreased appetite | No evidence of relapse |
| 5 | M | 80 | DLBCL | Fludarabine 30 mg/m² + cyclophosphamide 300 mg/m² on D−5, D−4, and D−3, followed by a 2-day rest period | Hepatitis B | Yes (prior to CAR-T therapy) | Yes  (prior to CAR-T therapy) | No | Oral candidiasis | Yes | Yes (grade 1) | Yes (grade 1) | Enteral feeding tube | No evidence of relapse |
| 6 | M | 42 | DLBCL | Fludarabine 25 mg/m² from D−5 to D−3 + cyclophosphamide 60 mg/kg on D−6. | None | Yes | No | No | No | No | No | No | General/nausea | Relapsed disease |
| 7 | M | 47 | B-ALL | Fludarabine 25 mg/m² from D−5 to D−3 and cyclophosphamide 60 mg/kg on D−6. | Diabetes mellitus | No | Yes | No | No | No | No | Yes (grade 1) | General | No evidence of relapse |
| 8 | M | 25 | B-ALL | Fludarabine 25 mg/m² from D−5 to D−3 and cyclophosphamide 60 mg/kg on D−6. | None | No | Yes | No | Yes (dental pain due to bruxism) | No | Yes (grade 1) | Yes (grade 2) | General/nausea | No evidence of relapse |
| 9 | M | 74 | MM | Fludarabine 30 mg/m² + cyclophosphamide 300 mg/m² on D−5, D−4, and D−3, followed by a 2-day rest period. | Hypothyroidism, hyperuricemia. | No | No | No | No | No | No | Yes (grade 1) | General | No evidence of relapse |
| 10 | M | 23 | B-ALL | Fludarabine 25 mg/m² from D−5 to D−3 + cyclophosphamide 60 mg/kg on D−6. | None | No | Yes | No | No | No | Yes (grade 4) | Yes (grade 1) | General | No evidence of relapse |
